# Particulate Mycobacterial Vaccines Induce Protective Immunity against Tuberculosis in Mice

**DOI:** 10.3390/nano11082060

**Published:** 2021-08-13

**Authors:** Shuxiong Chen, Diana H. Quan, Xiaonan T. Wang, Sarah Sandford, Joanna R. Kirman, Warwick J. Britton, Bernd H. A. Rehm

**Affiliations:** 1 Centre for Cell Factories and Biopolymers, Griffith Institute for Drug Discovery, Griffith University, Brisbane, QLD 4111, Australia; shuxiong.chen@griffith.edu.au; 2 Centenary Institute, The University of Sydney, Sydney, NSW 2050, Australia; d.quan@centenary.org.au (D.H.Q.); t.wang@centenary.org.au (X.T.W.); w.britton@centenary.org.au (W.J.B.); 3 The Peter Doherty Institute for Infection and Immunity, University of Melbourne, Melbourne, VIC 3000, Australia; ssandford@student.unimelb.edu.au; 4 Microbiology & Immunology Department, University of Otago, Dunedin 9016, New Zealand; jo.kirman@otago.ac.nz; 5 Department of Clinical Immunology, Royal Prince Alfred Hospital, Sydney, NSW 2050, Australia; 6 Menzies Health Institute Queensland, Griffith University, Gold Coast, QLD 4222, Australia

**Keywords:** bioengineering, self-assembly, tuberculosis, particulate vaccines, polyester nanoparticle, antigen nanoparticles, protective immunity

## Abstract

Currently available vaccines fail to provide consistent protection against tuberculosis (TB). New, improved vaccines are urgently needed for controlling the disease. The mycobacterial antigen fusions H4 (Ag85B-TB10.4) and H28 (Ag85B-TB10.4-Rv2660c) have been shown to be very immunogenic and have been considered as potential candidates for TB vaccine development. However, soluble protein vaccines are often poorly immunogenic, but augmented immune responses can be induced when selected antigens are delivered in particulate form. This study investigated whether the mycobacterial antigen fusions H4 and H28 can induce protective immunity when assembled into particulate vaccines (polyester nanoparticle-H4, polyester nanoparticle-H28, H4 nanoparticles and H28 nanoparticles). The particulate mycobacterial vaccines were assembled inside an engineered endotoxin-free production strain of *Escherichia coli* at high yield. Vaccine nanoparticles were purified and induced long-lasting antigen-specific T cell responses and protective immunity in mice challenged by aerosol with virulent *Mycobacterium tuberculosis*. A significant reduction of *M. tuberculosis* CFU, up to 0.7-log_10_ protection, occurred in the lungs of mice immunized with particulate vaccines in comparison to placebo-vaccinated mice (*p* < 0.0001). Polyester nanoparticles displaying the mycobacterial antigen fusion H4 induced a similar level of protective immunity in the lung when compared to *M. bovis* bacillus Calmette-Guérin (BCG), the currently approved TB vaccine. The safe and immunogenic polyester nanoparticle-H4 vaccine is a promising subunit vaccine candidate, as it can be cost-effectively manufactured and efficiently induces protection against TB.

## 1. Introduction

Tuberculosis (TB) is a major health burden, as more than 2 billion people are infected with *Mycobacterium tuberculosis* (*M. tuberculosis*) worldwide. The World Health Organization (WHO) estimates that approximately 10 million people develop active TB and 1.4 million people die from TB every year, making TB the leading infectious disease killer [1]. The immunocompromised population has a much higher risk for developing active TB, and this includes people with HIV infection, malnutrition or diabetes [2,3,4]. The spread of drug-resistant forms of TB is a growing threat to global public health [5,6]. Developing interventions to prevent TB is a crucial global health priority [7,8]. However, a vaccine should be capable of effectively protecting against TB, including its drug-resistant forms [2]. The *Mycobacterium bovis* bacillus Calmette-Guérin (BCG) vaccine remains the only licensed vaccine to prevent against TB. However, BCG is partially effective in its ability to protect against the most common form of TB: pulmonary infection in adults [9,10,11]. Accelerating the development of effective and economic vaccines will be essential for the control of the TB epidemic. 

The current TB vaccine candidates in clinical trials are categorized into two groups, whole mycobacterial cell-derived vaccines and subunit vaccines [2]. Whole cell vaccines are further sub-divided into live-attenuated vaccines (e.g., BCG, VPM 1002 and MTBVAC) and fractionated/killed vaccines (e.g., RUTI, MIP, DAR-901 and *Mycobacterium vaccae*) derived from whole mycobacteria cells [2]. The live-attenuated mycobacterial vaccines possess strong immunogenicity; however, they have a potential safety issue, as they may cause progressive infection in immunocompromised recipients [2,8]. Fractionated/killed vaccines derived from whole mycobacteria cells pose no infectious risk to people but are much less immunogenic than live-attenuated vaccines and may require the administration of multiple doses [2,8,12]. In comparison to the whole mycobacterial cell-derived vaccines, subunit vaccines comprise defined immunogenic components of mycobacteria. This is generally considered a safe approach. Nevertheless, a main constraint of subunit vaccines is poor immunogenicity [2,12]. An approach to increase subunit vaccine efficacy is to immobilize mycobacterial antigens on the surface of particulate platforms [13,14]. A number of studies have demonstrated that antigens displayed on nanoparticles can enhance the immune response to the respective antigens and effectively induce a protective immune response against various pathogens, such as malaria [15] and *Neisseria menigitidis* [16].

The mycobacterial antigen fusions H4, containing Ag85B and TB10.4, and H28, containing the H4 backbone and the additional TB-latency antigen Rv2660c, are very immunogenic when delivered with potent adjuvants [17,18,19]. We previously showed that these two mycobacterial antigen fusions, H4 and H28, can be engineered to self-assemble into various particulate mycobacterial vaccines (polyester nanoparticle-H4, polyester nanoparticle-H28, H4 nanoparticles and H28 nanoparticles) *in E. coli* cells. These particulate mycobacterial vaccines showed increased immunogenicity with respect to antibody responses and cell-mediated immunity in mice when compared to their soluble counter parts [20,21]. We have investigated whether the mycobacterial fusions H4 and/or H28 delivered as defined polyester or protein nanoparticles could induce protective immunity in a mouse model of aerosol *M. tuberculosis* infection. 

## 2. Materials and Methods 

### 2.1. Bacterial Strains and Nanoparticle Production Condition 

Table 1 lists the bacterial strains and plasmids applied in this study. An endotoxin free strain, ClearColi BL21(DE3), was used as the host for nanoparticle production. The methods of producing polyester nanoparticles and protein nanoparticles in ClearColi BL21(DE3) were previously described [20,21]. In particular, PhaA, PhaB and PhaC are the three key enzymes required for the formation of polyester nanoparticles. Genes encoding PhaA/PhaB and PhaC enzymes were cloned into pMCS69 and pET-14b, respectively. Therefore, both pET-14b phaC-TB and pMCS69 (harbouring *phaA* and *phaB* genes) were transformed into the production strains, ClearColi BL21(DE3) for polyester nanoparticle formation. In addition, ClearColi BL21(DE3) harboring only pET-14b His6-TB gene is required for the formation of the protein nanoparticle. Approximately 15 mL of an overnight cell culture were added to 500 mL of LB broth containing 0.5% (wt/vol) NaCl, 1% (wt/vol) glucose and the appropriate antibiotics ampicillin (100 µg mL^−1^) and/or chloramphenicol (100 µg mL^−1^). The bacterial cell culture was grown at 37 °C at 200 rpm for approximately 3 h. IPTG was used to induce the cell culture for nanoparticle production when the OD600 of the bacterial culture was approximately 0.5. The cell culture incubation was continued at 25 °C (for polyester nanoparticle production) or 37 °C (for protein nanoparticle production) at 200 rpm for 48 h.

### 2.2. Vaccine Isolation and Sterilization 

Bacterial cells harboring various particulate vaccines were disrupted using a microfluidizer (Microfluidics; Westwood, MA, USA) and purified by centrifugation through successive washing. The isolation, purification and sterilization procedures of nanoparticle vaccines have been described previously [20,21]. 

The isolation and purification of soluble vaccines were previously described by Chen et al. [21]. Briefly, purified protein nanoparticles (His6-H4 nanoparticles and His6-H28 nanoparticles) were solubilized using 8 M urea dissolved in Tris buffer (10 × 10^−3^ M Tris, pH7.5). The solubilized protein suspension was centrifugated and then filtered by 0.2 µm filter. The soluble protein solution was dialyzed three times to remove the urea using Tris buffer (10 × 10^−3^ M Tris, pH7.5). The solution containing soluble proteins was then centrifuged at 20,000× *g* for 30 min and subsequently sterilized using 0.2 µm filter. 

### 2.3. Nanoparticle Analysis and Immobilized Antigen Concentration Measurement

A 10% Bis-Tris gel was used to analyze the protein profile of purified protein nanoparticles. Densitometry analysis (Figure S2) of the protein gel was applied to evaluate a fusion protein purity of the total protein in 10% nanoparticle fractions. The analysis was performed using Image Lab Software (Bio-Rad Laboratories; Irvine, CA, USA). The amount of fusion protein was determined against a BSA standard curve. Cells producing nanoparticles and purified nanoparticles were analyzed using transmission electron microscopy (TEM). 

### 2.4. Vaccine Formulation

All the vaccines were emulsified in dimethyldioctadecyl ammonium bromide (DDA; MilliporeSigma, Burlington, MA, USA). The stock DDA solution at a concentration of 10 mg mL^−1^ was firstly prepared using sterile 10 mM Tris. HCl buffer pH 7.5. In particular, DDA powder was gently mixed with the sterile Tris buffer in a heated water bath at 80 °C. After dissolving, the DDA solution was cooled down at ambient temperature (approximately 25 °C) and then stored in a fridge at 4 °C. The final DDA solution showed a homogeneous white color. 

Both the particulate mycobacterial vaccines and the soluble forms, H4 and H28 recombinant fusions, were manufactured in an endotoxin free *E. coli* production host, ClearColi BL21(DE3). The formulated vaccines were comprised of 2–10 μg of mycobacterial antigens/dose, emulsified in 250 μg DDA/dose in a volume of 200 μL 10 mM Tris buffer pH7.5. The DDA alone was prepared in 10 mM Tris buffer was used as the negative control for both memory response and challenge studies. Vaccine samples were formulated with the DDA solution freshly before vaccination. *M. tuberculosis* culture filtrate protein (CFP) was obtained from the BEI Resources (Manassas, VA, USA ), and 10 µg were present in the DDA formulation used for immunization. Mice were vaccinated subcutaneously with formulated vaccines in a volume of 200 μL. 

### 2.5. Memory Response Study

The immunological memory study was approved by the Otago University Animal Ethics Committee (approval number 50-2016; see Institutional Review Board Statement). Female C57BL/6 mice (6–8 weeks of age) were initially bought from Jackson Laboratories (Bar Harbor, ME, USA) and then bred at the Biomedical Research Facility at the University of Otago. Ventilated cages in a specific pathogen free facility were used to individually maintain mice. 

Female C57BL/6 mice (*n* = 8) were vaccinated 3 times subcutaneously with mycobacterial H28 vaccines (soluble H28, polyester nanoparticles-H28 and H28 nanoparticles) formulated with adjuvant DDA at 9-day intervals. Half of the mice were euthanized 3 weeks after the final vaccination. The spleen tissues were harvested and prepared for a splenocyte restimulation assay using soluble H28. Another half of the mice were culled 12 weeks later after the final vaccination for memory response evaluation by analyzing in vitro cytokine release from the splenocyte. 

For measuring in vitro memory responses, a 70 µm cell strainer (Corning; NY, USA) was used to prepare the single cell suspension. After two washes with incomplete RPMI medium (Life Technologies; NY, USA) supplemented with penicillin (100 U mL^−1^; Life Technologies; USA) and streptomycin (100 U mL^−1^; Life Technologies; USA), red blood cells were removed by treating the cell suspension with red blood cell lysis buffer (Sigma-Aldrich; MO, USA). The cell suspensions were then washed and re-suspended in complete RPMI (Life Technologies; USA) supplemented with penicillin (100 U/mL), streptomycin (100 U/mL) and 5% (vol/vol) fetal calf serum (Life Technologies; USA). Trypan blue (1:100) was used to stain cells and a live cell count was performed using a hemocytometer. 

The splenocyte restimulation was described previously [20,21]. Briefly, 100 μL of single spleen cells suspensions (at a concentration of 5 × 10^6^ per mL, diluted with complete RPMI media) were loaded to U-bottomed 96-well plates (Life Technologies; USA). Subsequently, 100 µL of soluble His6-H28 antigen at a concentration of 40 µg/mL were added to plates to re-stimulate the cells. The incubation of the cell culture was carried out at 37 °C in 5% CO_2_ for 24 h or 60 h. Secreted cytokine in the supernatant fraction was measured using a BD CBA Mouse Th1/Th2/Th17 cytokine kit (BD Biosciences; CA, USA) according to the manufacturer’s instructions. 

### 2.6. Mycobacteria Growth Conditions

The *M. bovis* BCG Pasteur and *M. tuberculosis* H37Rv strain were cultured in Middlebrook 7H9 (Difco) medium, which was supplemented with glycerol (0.2% *v/v*), tyloxapol (0.02% *v/v*) and albumin-dextrose-catalase (ADC; 10% *v/v*). The mycobacterial strains were enumerated by plating serially diluted organ homogenates onto 7H11 (Difco) agar, containing oleic-acid-albumin-dextrose-catalase (OADC; 10% *v/v*) and glycerol (0.5% *v/v*).

### 2.7. M. tuberculosis Challenge Study

The Sydney Local Health District Animal Welfare Committee approved the *M. tuberculosis* infection experiments in accordance with relevant guidelines and regulations (approval number: 2020-003A). Female C57BL/6, approximately 6–8 weeks of age, was bought from the Animal Resources Centre (Perth, Australia) and housed under specific pathogen-free conditions. 

Female C57BL/6, approximately 6–8 weeks of age (*n* = 8), was immunized 3 times subcutaneously with TB vaccines (polyester nanoparticles, polyester nanoparticle-H4, polyester nanoparticles-H28, H4 nanoparticles, H28 nanoparticles, soluble H4 and soluble H28), or CFP formulated with DDA adjuvant at two-week intervals or vaccinated once with BCG. 

For the protection experiments, the mice were subcutaneously injected once with 5 × 10^5^ CFU of BCG Pasteur (200 µL in PBS) or immunized three times at two-week intervals with 10 μg/dose of H4, H28, polyester nanoparticle-H4, polyester nanoparticle-H28 or CFP formulated in 10 mM Tris buffer (pH 7.5) with 250 µg/dose of DDA in the volume of 200 µL. The mice administered with vehicle only were used as negative controls. For the challenge experiments, *M. tuberculosis* H37Rv with an infective dose of approximately 100 viable bacilli was used to infect mice 4 weeks after the last immunization via the aerosol route, using a Middlebrook airborne infection apparatus (Glas-Col; IN, USA). Six weeks after aerosol *M. tuberculosis* infection, the lung and spleen tissues were harvested and homogenized. The resulting suspensions from the lung and spleen tissue samples were plated after serial dilution on Middlebrook 7H11 agar plates containing 10% oleic acid-albumin-dextrose-catalase. The plates incubation was performed at 37 °C and CFUs were obtained approximately 3 weeks later. 

### 2.8. Statistical Analysis

GraphPad (Prism version 8) was used to evaluate the statistical significance of the differences between the experimental groups. This was analyzed by a one- or two-way analysis of variance (ANOVA), with pairwise comparison of multi-grouped data sets achieved using Tukey’s or Dunnett’s post hoc test.

## 3. Results

### 3.1. Production of Particulate Mycobacteiral Vaccines

**Figure 1:**
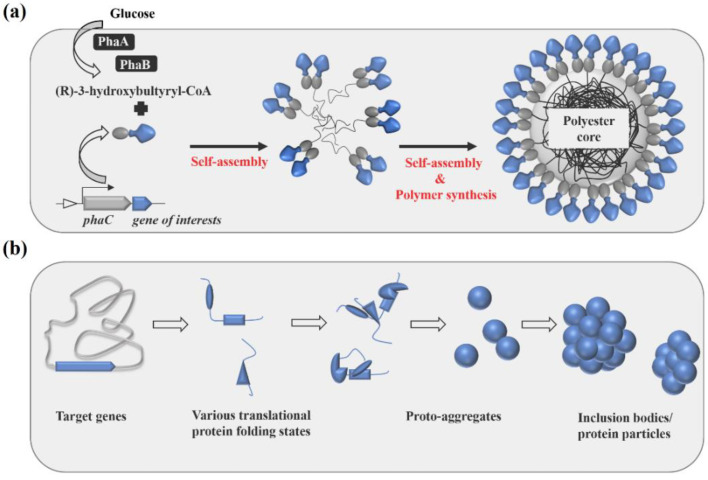
Schematic diagram of the in vivo assembly of antigen-coated polyester nanoparticles (**a**) and antigen nanoparticles (**b**).

**Figure 2:**
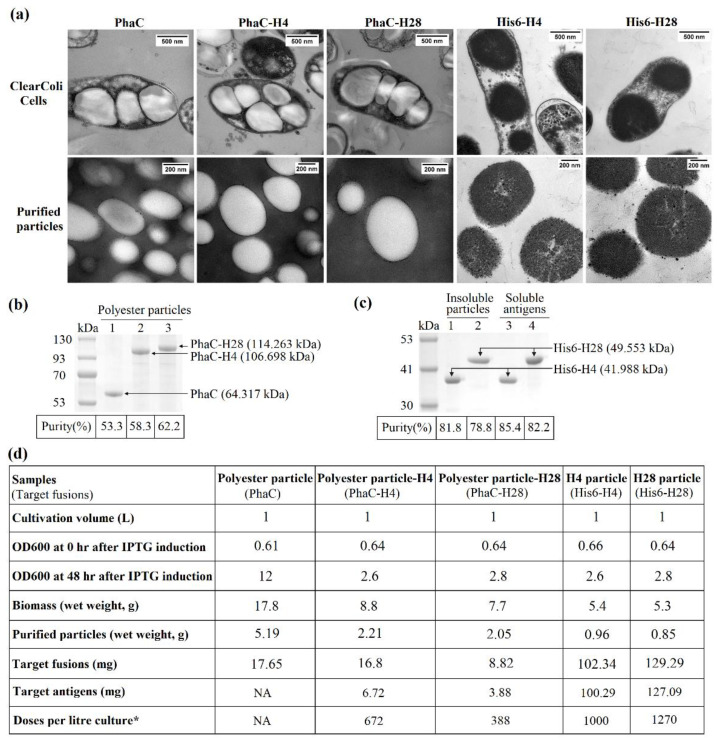
Vaccine nanoparticle production and characterization. (**a**) TEM images of ClearColi BL21 (DE3) producing various particulate mycobacterial vaccines and of purified particulate vaccines. (**b**) Protein profile of purified polyester nanoparticle-TB vaccines. kDa, MW marker (GangNam-Stain Prestained Protein Ladder); lane 1, PhaC (64.317 kDa); lane 2, PhaC-H4 (106.698 kDa); lane 3, PhaC-H28 (114.263 kDa). (**c**) Protein profile of mycobacterial H4 and H28 antigen nanoparticles. kDa, MW marker (GangNam-Stain Prestained Protein Ladder); lanes 1 and 3, His6-H4 (41.988 kDa); lanes 2 and 4, His6-H28 (49.553 kDa). (**d**) Composition and yield of particulate mycobacterial vaccines. * The total number of doses per liter culture was calculated using the highest dose administered, 10µg/dose.

Recombinant genes were constructed to encode fusion proteins that mediated the in vivo self-assembly of the mycobacterial particulate vaccines (Table 1). To produce polyester nanoparticle-TB vaccines (Figure 1a), the pET-14b plasmid containing the respective hybrid genes was introduced into an endotoxin free production host, ClearColi BL21(DE3) harboring pMCS69, containing the genes encoding enzymes PhaA and PhaB. These two enzymes provided the precursor molecules to PhaC enzyme for the synthesis of poly(3-hydroxybutyric acid), which has been extensively studied and exploited in the medical field due to its properties, such as its biodegradability and biocompatibility [14,23,24]. The PhaC enzyme remains covalently attached on the polyester nanoparticle surface after nanoparticle formation [20,23]. The selected mycobacterial vaccines H4 and H28 were translationally fused to the C-terminus of PhaC, which acts as polyester nanoparticle protein anchor. The in vivo formation of polyester nanoparticles-TB vaccines in ClearColi BL21(DE3) was confirmed by TEM, and the size of the produced polyester nanoparticle is between 200 and 800 nm (Figure 2a and Figure S1c). The protein profile of full-length PhaC-mycobacterial antigen fusions in the nanoparticle fractions (Figure 2b and Figure S1a) was analyzed on a 10% Bis-Tris gel to investigate whether the mycobacterial antigens were displayed on the nanoparticle surface. The PhaC-mycobacterial antigen fusions were overproduced because the molecular weights (MWs) of the dominant fusion proteins corresponded to the theoretical MWs of PhaC (64.317 kDa), PhaC-H4 (106.698 kDa) and PhaC-H28 (114.263 kDa), suggesting the mycobacterial antigens were successfully immobilized on the nanoparticle surface through the polyester anchor PhaC. The densitometry results on the Bis-Tris gel showed that the PhaC-H4 and PhaC-H28 vaccines accounted for 58.3% and 62.2% of the total protein in the PhaC-H4 and PhaC-H28 nanoparticle fractions, respectively. The production of the polyester nanoparticle displaying the mycobacterial vaccines in *E. coli* cells achieved a high yield of >25% of biomass.

To mediate the intracellular self-assembly of mycobacterial antigen nanoparticles/inclusion bodies (His6-H4 and His6-H28 nanoparticles) in ClearColi BL21(DE3) cells, recombinant genes encoding His6-H4 and His6-H28 fusion proteins were introduced into the pET-14b expression vector harboring the strong T7 promoter for overproduction of recombinant protein towards the formation of inclusion bodies (Figure 1b). The addition of His6 tag to the N-terminus of H4 and/or H28 was to stimulate overproduction of H4 and/or H28 proteins, leading to the inclusion bodies/nanoparticle formation, as the positively charged His6 may alter the intrinsic properties, such as the net charge or correct folding capacity, of the target proteins H4 and/or H28 [21]. The TEM study showed that the mycobacterial antigen nanoparticles were produced abundantly in ClearColi BL21(DE3) cells, the purified antigen nanoparticles were in an oval shape and the size ranged between 200 and 800 nm (Figure 2a). The protein profile of the purified antigen nanoparticles on Bis-tris gel showed the mycobacterial recombinant fusion bands (His6-H4 and His6-H28) accounted for approximately 80% of the total protein in the antigen nanoparticle fractions (H4 and H28 nanoparticles), indicating the high purity of the vaccine samples (Figure 2c and Figure S1b). A high yield of the mycobacterial antigen nanoparticles (H4 or H28 nanoparticles), approximately 17% of the biomass, was obtained in the recombinant *E. coli* cells. 

### 3.2. Memory Response Evaluation

**Figure 3:**
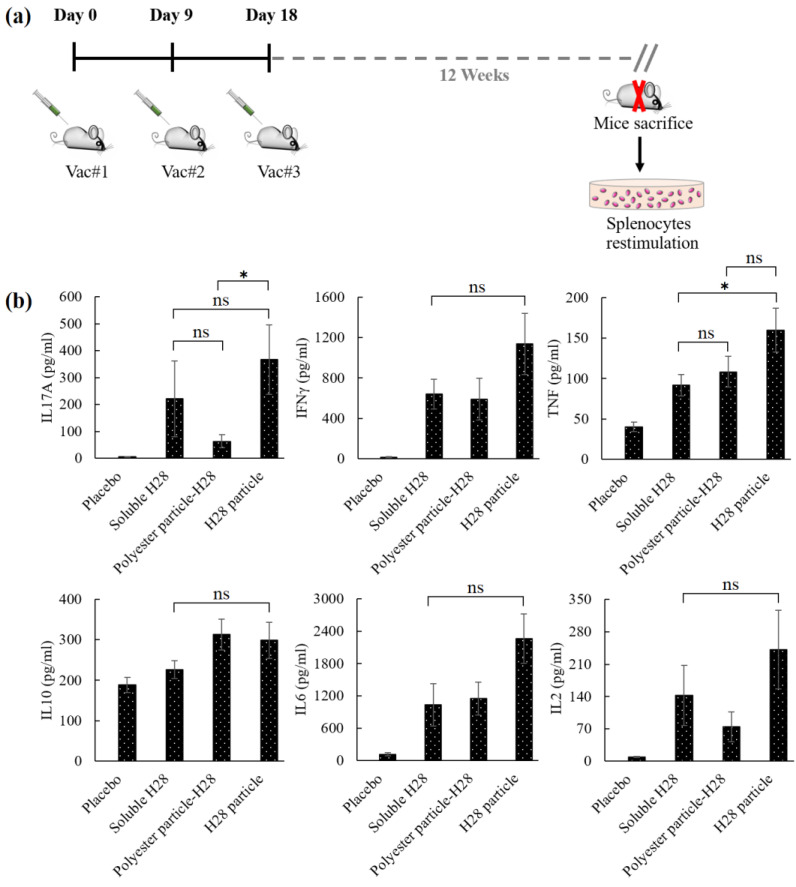
Induction of cytokines by particulate vaccines. (**a**) Timeline of the immunization schedule and memory response study. (**b**) Mice were sacrificed 12 weeks after the final vaccination. Murine splenocytes were cultured for 24 h with soluble H28. The release of cytokines was determined by a cytometric bead array. Each data point represents the mean for 8 mice ± the standard error of the mean. *, significant difference between experimental groups (*p* < 0.05); ns, no significant difference between experimental groups (*p* > 0.05).

The mycobacterial vaccine H28 is comprised of the vaccine H4 (Ag85B-TB10.4) backbone and the latency-associated antigen Rv2660c [2,25]. Thus, the memory study was performed in mice immunized with H28 vaccines (H28 nanoparticles, polyester nanoparticle-H28 and soluble H28), which were sacrificed at 12 weeks after the final vaccination. We measured the memory response by analyzing the in vitro cytokine secretion from their splenocytes re-stimulated with soluble H28. Cytokines were measured at early (24 h, Figure 3b) and late (60 h, Figure S3) time points after the splenocyte restimulation to allow for the observation of cytokines that were secreted and/or consumed later during the culture. 

Mice immunized with various H28 vaccines (H28 nanoparticles, polyester nanoparticle-H28 and soluble H28) showed higher cytokine secretion 12 weeks after the final vaccination when compared with the placebo mice, which were vaccinated with DDA adjuvant alone (Figure 3 and Figure S3). This indicated that the various H28 vaccines induced a strong memory response in mice evidenced by strong cytokine secretion. Interferon gamma (IFNγ) secretion by splenocytes from H28 nanoparticle-immunized mice was relatively high in comparison to soluble H28 and polyester nanoparticle-H28 vaccinated mice, but it was not statistically significant (Figure 3b). In addition, the secretion of interleukin 10 (IL10), interleukin 6 (IL6) and interleukin 2 (IL2) from mice immunized with various H28 vaccines showed no significant difference (Figure 3b).

Interleukin 17A (IL17A) production in soluble H28 immunized mice had no statistical difference with polyester nanoparticle-H28 or H28 nanoparticle vaccinated mice (*p* < 0.05). However, IL17A production was significantly higher in the H28 nanoparticle immunized group than in the polyester nanoparticle-H28 vaccinated group (Figure 3b) (*p* < 0.05). There was no significant difference between the tumor necrosis factor alpha (TNFα) production in the mice immunized with soluble H28 and that in the polyester nanoparticle-H28 group (Figure 3b). TNFα production was significantly higher in the H28 nanoparticle-immunized mice than in the polyester nanoparticle-H28 vaccinated group (*p* < 0.05). Accordingly, mycobacterial H28 vaccines generated robust recall responses in mice up to 12 weeks after the final booster vaccination. 

### 3.3. M. Tuberculosis Infection Study

**Figure 4:**
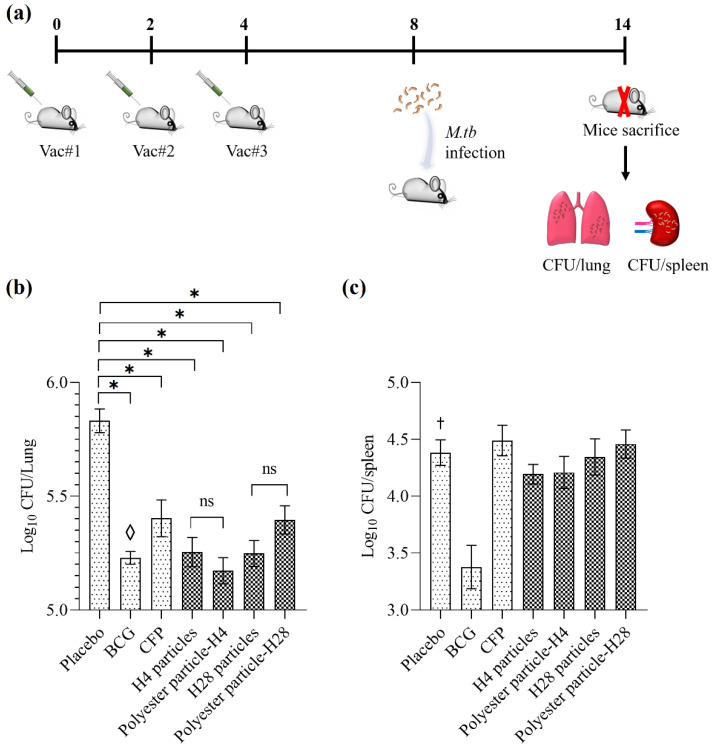
Protective immunity induced by particulate vaccines against infection with *M. tuberculosis*. (**a**) Schematic diagram of the mice immunization plan and *M. tuberculosis* infection. (**b**) *M. tuberculosis* CFUs in the lungs of mice vaccinated with 5 × 10^5^ BCG, 5 µg *M. tuberculosis* CFP, H4 nanoparticles, polyester nanoparticles-H4, H28 nanoparticles and polyester nanoparticles-H28. (**c**) *M. tuberculosis* CFUs in the spleens of vaccinated mice. Data are shown as the means for 8 mice ± the standard error of the mean. Statistical significances were determined by one-way ANOVA with a pairwise comparison of multi-grouped data sets using Dunnett’s post hoc test. *, significant difference between all vaccinated groups and the placebo group that received DDA alone (*p* < 0.0001); ◊, no significant difference between BCG and the other vaccinated groups. †, significant difference between the placebo group and BCG (*p* < 0.0001), but no significant difference with the other vaccinated groups; ns, no significant difference (*p* > 0.05).

For the challenge study, the mice were immunized with various mycobacterial vaccines (H4 nanoparticles, polyester nanoparticles-H4, H28 nanoparticles and polyester nanoparticles-H28) and infected with aerosol *M. tuberculosis* H37Rv 4 (100 CFU/mouse) weeks after the last boost (Figure 4a). The lung and spleen tissues of the individual mice were processed 6 weeks after the infection in order to determine the bacterial counts in the respective tissues (Figure 4a). 

There was a significant reduction of CFU counts in the lungs of mice receiving the particulate mycobacterial vaccines (H4 nanoparticles, polyester nanoparticles-H4, H28 nanoparticles and polyester nanoparticles-H28), CFP and BCG compared to the Tris buffer-immunized negative control, placebo group (Figure 4b) (*p* < 0.0001). There was no significant difference in the lung CFU counts between the mice immunized with polyester nanoparticle-H4 and those immunized with H4 nanoparticles. The same observation was also found in the mice vaccinated with polyester nanoparticle-H28 and H28 nanoparticles (Figure 4b) (*p* > 0.05). Encouragingly, the CFU counts in the lungs of the mice immunized with BCG showed no significant differences with the other vaccinated groups (H4 nanoparticles, polyester nanoparticles-H4, H28 nanoparticles, polyester nanoparticles-H28, and CFP) (Figure 4b) (*p* > 0.05). However, the mice vaccinated with particulate mycobacterial vaccines showed no significant reduction in CFU counts in the spleen compared to the negative placebo group (*p* > 0.05). 

## 4. Discussion

This study demonstrated that the mycobacterial antigen fusions H4 and H28 displayed on polyester nanoparticles or assembled into nanoparticles can induce long-lasting antigen-specific T cell memory responses and generate protective immunity in the lungs of mice after aerosol *M. tuberculosis* infection. Encouragingly, immunization with polyester nanoparticles displaying the mycobacterial vaccine H4 induced comparable protective immunity in the lungs of mice as BCG immunization after aerosol *M. tuberculosis* infection.

Particulate vaccine delivery systems such as antigen-coated polyester nanoparticles and antigen nanoparticles produced inside *E. coli* cells have been previously described, along with their capacity to induce specific and strong immune responses to mycobacterial antigens [13,20,21,23,26]. All particulate mycobacterial vaccines used in this study were produced in the endotoxin-free production host, ClearColi BL21 (DE3), in which genes encoding lipopolysaccharide biosynthesis genes have been knocked out [27]. Previous studies showed that the preparation of a soluble mycobacterial vaccine required laborious chromatography purification steps [28]. However, the particulate vaccine isolation and purification procedures employed in this study were simple and cost-effective when compared to conventional protein purification, as only washing steps using centrifugation were required after cell disruption. In addition, recombinant plasmid constructs mediated the one-step production of mycobacterial nanoparticle vaccines at a high purity and yield. An earlier study also showed that polyester nanoparticles displaying carbonic anhydrase retained proper enzymatic activity of up to 90 °C [29], indicating that the polyester nanoparticle platform possesses intrinsic thermostability.

Some of the background/contaminating proteins from *E. coli* were co-purified with the particulate vaccines after the nanoparticle extraction (Figure 2b,c, and Figure S1a,b). Studies showed that mice immunized with various particulate TB vaccines (polyester nanoparticle-H4, polyester nanoparticle-H28, H4 nanoparticles and H28 nanoparticles) only generated specific antibodies to mycobacterial vaccines H4 and/or H28 and had no antibody response to the host-cell-protein impurities [20,21]. This suggested that only the antigen (H4 and H28)-specific antibody responses were generated by the particulate TB vaccines. Polyester nanoparticle-TB vaccines comprise a hydrophobic polyester core covalently coated with mycobacterial antigens via the anchoring protein PhaC. Several studies have demonstrated that the serum samples from mice immunized with polyester nanoparticles with/without antigens do not interact with the plain polyester nanoparticles. In addition, the in vitro splenocyte re-stimulation assay showed that the plain polyester nanoparticles cannot stimulate T cell responses [13,20,23]. This suggests that polyester nanoparticles and/or PhaC are poorly immunogenic and unable to induce a detectable immune response in mice. 

In this study, the immunological memory response was analyzed by comparing the cytokine secretion of splenocytes obtained from various H28 vaccines (H28 nanoparticles and polyester nanoparticle-H28)-immunized mice at 12 weeks after the final vaccination following in vitro 24 h (Figure 3) and 60 h (Figure S3) re-stimulation with vaccine antigens. The purpose of measuring the cytokine secretion at different time points was to analyze the cytokine consumption by cells during the re-stimulation process. For example, IL2 plays an important role in T cell proliferation and differentiation. IL2 secretion was between 70–250 pg/mL at the early time point (24 h; Figure 3); however, it was quickly depleted at the late time point (60 h; Figure S3). Cell-mediated immunity driven by polyfunctional CD4+ T cells plays an important role in the eradication of the intracellular pathogen *M. tuberculosis* [30,31]. Splenocytes from mice immunized with polyester nanoparticles-H28 and mycobacterial H28 nanoparticles taken at 3 weeks after the final vaccination induced the strong production of Th1 cytokines (IFNγ, IL2 and TNF-α) upon soluble H28 re-stimulation when compared to the placebo group [20,21]. Further, there was a persistence of the strong Th1 cytokine response, as cytokine release was still observed in splenocytes from mice taken 12 weeks after the final vaccination. This demonstrates that TB vaccines delivered in particulate form attached to polyester nanoparticles or assembled into nanoparticles are capable of inducing long-lived T cell memory responses after vaccination. 

The induction of strong immunogenicity after vaccination does not always corelate with protective immune responses [32,33,34,35]. Moreover, both IFNγ-deficient and wild-type mice respond to BCG vaccination with similar reduction in the bacterial burden after *M. tuberculosis* infection [36]. Thus, the secretion of Th1 cytokines by polyfunctional memory CD4+ T cells may not be required to improve protective immunity against mycobacterial pathogens. The failure to develop more effective TB vaccines over the past decades might be due to the failure to pay sufficient attention to the induction and persistence of memory T cell responses [22,32,34]. Furthermore, recent findings suggest that antibody responses may also be important mediators of vaccine-induced protective immunity against TB [32,34]. Since our particulate mycobacterial vaccines (polyester nanoparticle-H4, polyester nanoparticle-H28, H4 nanoparticles, and H28 nanoparticles) induced strong and specific antibodies to the soluble mycobacterial vaccine antigens, this could further underpin vaccine performance [20,21]. 

This study showed that the particulate vaccines (H4 nanoparticles, polyester nanoparticles-H4, H28 nanoparticles and polyester nanoparticles-H28) induced an immune response that correlated with a significant reduction in the *M. tuberculosis* bacterial load in the lungs compared to the negative control mice (Figure 4). In contrast to vaccine H4, H28 contains an additional latency-associated antigen Rv2660c. However, the addition of this latency antigen did not increase the protective efficacy of H28 particulate vaccines. There was trend for the polyester particulate H4 vaccines to provide increased protection compared to the polyester particulate H28 vaccines (Figure 4). This finding is consistent with a previous study that found that soluble H4 induced a better protective immunity than soluble H28 in various animal models [28,37,38,39,40]. Soluble H4 can lead to a significant reduction of CFU counts in both the lungs and spleen, but soluble H28 was not able to significantly reduce the bacterial load in the spleen (Figure S4b). Furthermore, Figure 4b shows that all of the nanoparticle vaccine-immunized mice as well as the BCG and CFP-vaccinated groups were able to provide protection in the lungs, as they led to a significant reduction of CFU compared to the placebo. However, only BCG can greatly reduce the CFU counts in the spleen compared to other vaccinated groups (CFP, H4 nanoparticles, polyester nanoparticles-H4, H28 nanoparticles and polyester nanoparticles-H28) (Figure 4c). A similar finding was observed in a previous study [26]. This may be because only the BCG is a live-attenuated vaccine. The others are subunit vaccines, which are unable to replicate and thus possess a limited capability of inducing a strong and protective immunity against extrapulmonary TB [14,26]. In addition, immunization with H4 vaccines (including soluble H4, polyester nanoparticle-H4 and H4 nanoparticles) resulted in a similar level of protection in the lungs as the BCG immunization of mice after *M. tuberculosis* infection (Figure 4b and Figure S4a). However, the manufacturing of particulate vaccines (polyester nanoparticle-H4 and H4 nanoparticles) is more cost-effective compared to soluble H4, as they do not need laborious purification procedures and only require simple washings via centrifugation. Although there was no significant difference in the CFU counts in the lungs of mice immunized with H4 nanoparticles and polyester nanoparticles-H4, H4 nanoparticles were instable, as approximately 6 months after H4 nanoparticle purification, the full-length of H4 band can be observed on 10% Bis-Tris gel but cannot be detected by an immunoblot assay using an anti-H4 antibody (data not shown). Accordingly, the mycobacterial H4 vaccine displayed on polyester nanoparticles can perform as an efficacious and stable TB vaccine candidate by inducing protective immunity against *M. tuberculosis*. 

## 5. Conclusions

Particulate mycobacterial vaccines (polyester nanoparticle-H4, polyester nanoparticle-H28, H4 nanoparticles and H28 nanoparticles) were produced successfully in the engineered endotoxin-free production strain ClearColi BL21 (DE3) at a high yield, enabling the isolation of purified vaccine nanoparticles. These particulate vaccines exhibited superior immunological properties when compared to their soluble counterpart. The vaccine nanoparticles were safe and stable at 4 °C. The particulate vaccines induced long-lasting memory response. Promisingly, all particulate vaccines generated protective immunity in the lung after aerosol *M. tuberculosis* infection. In particular, the mycobacterial vaccine H4 displayed on a polyester nanoparticle showed a similar level of protection in the lungs as the BCG following aerosol *M. tuberculosis* infection. It should be noted that BCG, in contrast to subunit vaccines, is a live attenuated vaccine that is able to replicate, which enhances the induction of strong immune responses and protective immunity against pulmonary TB. However, the polyester nanoparticle based H4 vaccine is a promising vaccine candidate, as it can be cost-effectively produced in one-step using engineered *E. coli* and induces protective immunity against this major human pathogen to a degree comparable to the current BCG vaccine. 

## Figures and Tables

**Table 1:** Bacterial production strains and relevant plasmids applied in this study.

**Strains or Plasmids**	**Characteristics ^a^**	**Sources or References**
***E. coli***		
ClearColi BL21 (DE3)	F^–^ *ompT hsdS_B_ (r_B_^-^ m_B_^-^) gal dcm lon* λ(DE3 [*lacI lac*UV5-T7 gene 1 *ind1 sam7 nin5*]) *msbA148* ∆*gutQ* ∆*kdsD* ∆*lpxL* ∆*lpxM ∆pagP* ∆*lpxP* ∆*eptA*	Lucigen
**Plasmids**		
pET-14b	Amp^R^; T7 promoter	Novagen
pET-14b PhaC	pET-14b containing fragment gene *phaC*	[20]
pET-14b PhaC-H4	pET-14b consisting of *Bam*HI gene fragment *h4* introduced to the 3’ end of *phaC*	[20]
pET-14b PhaC-H28	pET-14b consisting of *Bam*HI gene fragment *h28* introduced to the 3´ end of *phaC*	[20]
pET-14b His6-H4	pET-14b consisting of *Nde*I/*Bam*HI fragment gene *his6-h4*	[21]
pET-14b His6-H28	pET-14b consisting of *Nde*I/*Bam*HI fragment gene *his6-h28*	[21]
pMCS69	Cm^R^; T7 promoter, pBBR1MCS derivative consisting of genes *phaA* and *phaB*, from *C. necator*, colinear to *lac* promoter	[22]

^a^ Cm^R^, chloramphenicol resistance; Amp^R^, ampicillin resistance; *h4*, *ag85b-tb10.4*; *h28*, *Ag85B-TB10.4-Rv2660c.*

## Data Availability

The datasets of this study are available from the corresponding author on reasonable request. All recorded raw data are archived at Griffith Institute for Drug Discovery, Griffith University.

## Supplementary Materials

The following are available online at https://www.mdpi.com/article/10.3390/nano11082060/s1: Figure S1: full protein profile of purified mycobacterial vaccine samples; Figure S2: mycobacterial antigen concentration measurement; Figure S3: cytokine release by murine splenocytes, harvested 12 weeks after the final vaccination with mycobacterial H28 vaccines, following 60 h restimulation with soluble H28; Figure S4: *M. tuberculosis* burden in the lungs from mice immunized with the soluble TB vaccines following *M. tuberculosis* infection.
