# Supplementary material for: Particulate Mycobacterial Vaccines Induce Protective Immunity against Tuberculosis in Mice
Source: Nanomaterials (Basel). 2021 Aug 13;11(8):2060. doi: 10.3390/nano11082060 (PMC8402087; doi:10.3390/nano11082060)
Supplement: Supplementary file 1 [file nanomaterials-11-02060-s001.zip › nanomaterials-1299720-supplementary.pdf]

Supplementary Material

Particulate Mycobacterial Vaccines Induce Protective Immunity against Tuberculosis in Mice

Shuxiong Chen [1], Diana H. Quan [2], Xiaonan T. Wang [2], Sarah Sandford [3], Joanna R. Kirman [4], Warwick J. Britton [2,5] and Bernd H. A. Rehm [1,6,*]

[1] Centre for Cell Factories and Biopolymers, Griffith Institute for Drug Discovery, Griffith University, Brisbane, QLD 4111, Australia; shuxiong.chen@griffith.edu.au
[2] Centenary Institute, The University of Sydney, Sydney, NSW 2050, Australia; d.quan@centenary.org.au (D.H.Q.); t.wang@centenary.org.au (X.T.W.); w.britton@centenary.org.au (W.J.B.)
[3] The Peter Doherty Institute for Infection and Immunity, University of Melbourne, Melbourne, VIC 3000, Australia; ssandford@student.unimelb.edu.au
[4] Microbiology & Immunology Department, University of Otago, Dunedin 9016, New Zealand; jo.kirman@otago.ac.nz
[5] Department of Clinical Immunology, Royal Prince Alfred Hospital, Sydney, NSW 2050, Australia
[6] Menzies Health Institute Queensland, Griffith University, Gold Coast, QLD 4222, Australia
* Correspondence: b.rehm@griffith.edu.au; Tel.: +61-(0)7-3735-4233

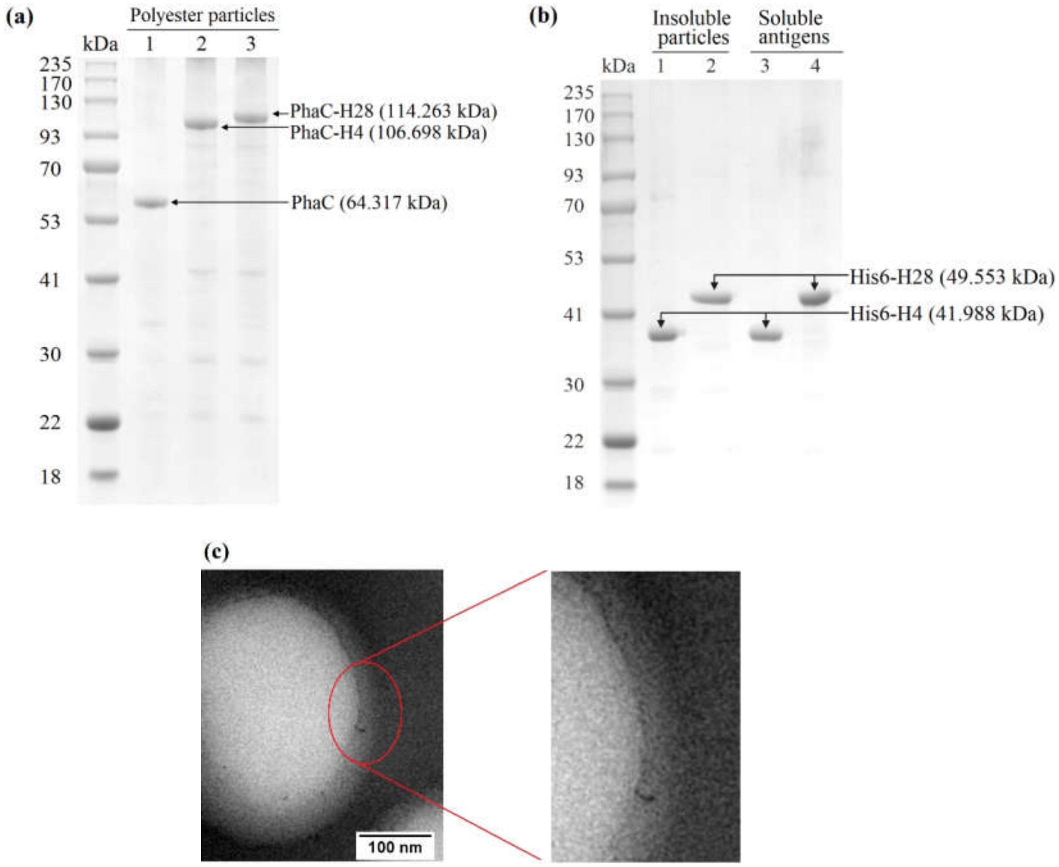

Figure S1. Full protein profile of purified mycobacterial vaccine samples. (**a**) Protein profile of purified polyester particle-TB vaccines. kDa, MW marker (GangNam-Stain Prestained Protein Ladder); lane 1, PhaC (64.317 kDa); lane 2, PhaC-H4 (106.698 kDa); lane 3, PhaC-H28 (114.263 kDa). (**b**) Protein profile of purified mycobacterial antigen particles. kDa, MW marker (GangNam-Stain Prestained Protein Ladder); lanes 1 and 3, His6-H4 (41.988 kDa); lanes 2 and 4, His6-H28 (49.553 kDa). (**c**) TEM image of purified polyester particle produced from ClearColi BL21 (DE3) harbouring pET-14b phaC and pMCS69. The polyester particle consists of polyester core (white) surrounded by abundant layer of polyester synthase PhaC (red circle).

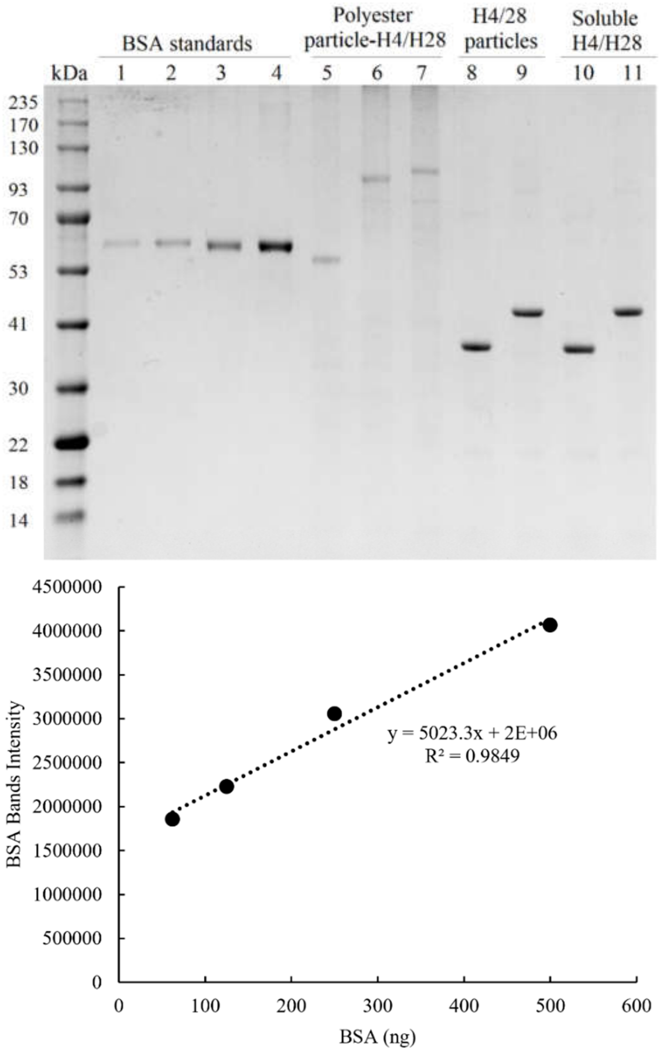

Figure S2. Mycobacterial antigen concentration measurement. Various BSA standards, 62.5 ng, 125 ng, 250 g, and 500 ng, were loaded on Bis-Tris gel to generate a standard curve for determination of mycobacterial antigen H4/H28 concentration in the 10% particle slurry. The Bis-Tris gel image was taken by the gel doc (BioRad Laborotories, Hercules, CA). The band intensity of BSA standards and recombinant TB antigens was analysed by the Image Lab Software (BioRad Laborotories, Hercules, CA).

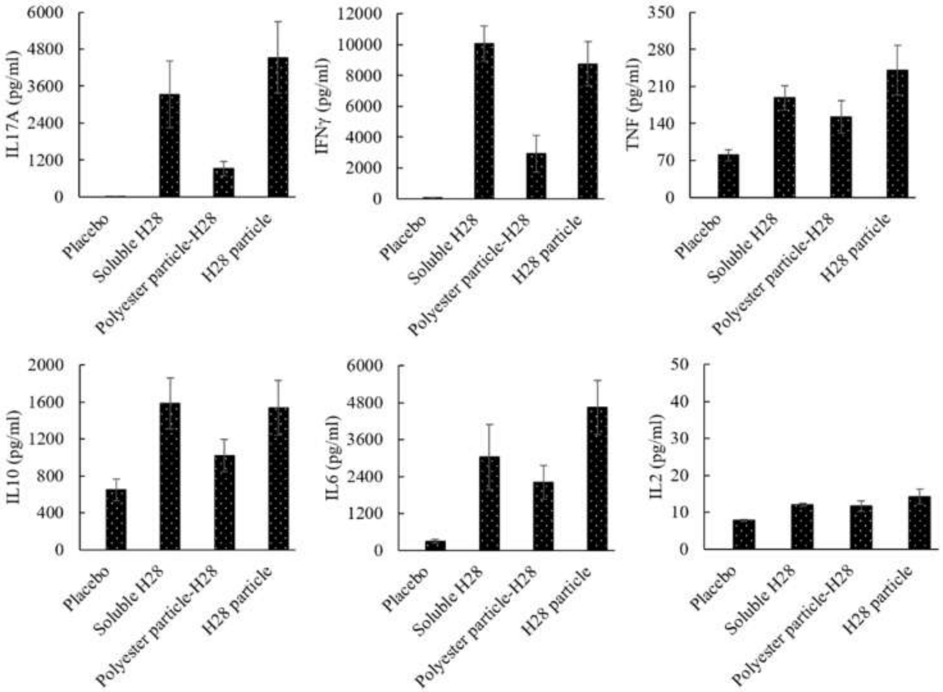

Figure S3. Cytokine release by murine splenocytes, harvested 12 weeks later after the final vaccination with mycobacterial H28 vaccines, following 60 h restimulation with soluble H28. Mice were culled 12 weeks later after the final vaccination. Murine splenocytes were incubated for 60 h with soluble H28. The release of cytokines was measured by cytometric bead array. Each data point stands for the mean for 8 mice ± the standard error of the mean.

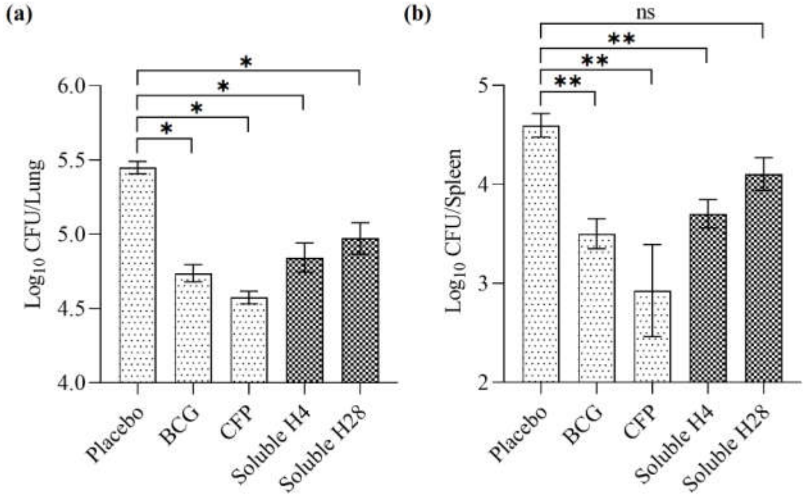

Figure S4. *M. tuberculosis* burden in the lungs and spleens from mice immunized with the soluble TB vaccines following *M. tuberculosis* infection. A. *M. tuberculosis* CFU in the lungs of mice vaccinated with BCG as well as soluble H4, H28 and *M. tuberculosis* CFP in DDA vaccines. B. CFU counts in the spleens of immunized mice. Each Histogram represents for the mean for 8 mice ± the standard error of the mean. *, significant difference between all vaccinated groups and placebo (DDA alone) ($p < 0.001$). **, significant difference between vaccinated groups and the placebo group that received DDA alone ($p < 0.05$); ns, no significant difference between soluble H28 vaccinated group and the placebo group ($p > 0.05$).
